# Association of Low Baseline Diaphragm Muscle Mass With Prolonged Mechanical Ventilation and Mortality Among Critically Ill Adults

**DOI:** 10.1001/jamanetworkopen.2019.21520

**Published:** 2020-02-19

**Authors:** Michael C. Sklar, Martin Dres, Eddy Fan, Gordon D. Rubenfeld, Damon C. Scales, Margaret S. Herridge, Nuttapol Rittayamai, Michael O. Harhay, W. Darlene Reid, George Tomlinson, Dmitry Rozenberg, William McClelland, Stephen Riegler, Arthur S. Slutsky, Laurent Brochard, Niall D. Ferguson, Ewan C. Goligher

**Affiliations:** 1 Interdepartmental Division of Critical Care Medicine, University of Toronto, Toronto, Ontario, Canada; 2 Keenan Centre for Biomedical Research, Li Ka Shing Knowledge Institute, St Michael’s Hospital, Toronto, Ontario, Canada; 3 AP-HP, Service de Pneumologie, Médecine Intensive–Réanimation (Département “R3S”), Groupe Hospitalier Pitié-Salpêtrière Charles Foix, Paris, France; 4 Institute for Health Policy, Management, and Evaluation, University of Toronto, Toronto, Ontario, Canada; 5 Division of Respirology, Department of Medicine, University Health Network, Toronto, Ontario, Canada; 6 Department of Critical Care Medicine, Sunnybrook Health Science Centre, Toronto, Ontario, Canada; 7 Toronto General Hospital Research Institute, Toronto, Ontario, Canada; 8 Siriraj Hospital, Division of Respiratory Disease and Tuberculosis, Department of Medicine, Faculty of Medicine, Mahidol University, Bangkok, Thailand; 9 Palliative and Advanced Illness Research (PAIR) Center, Perelman School of Medicine, University of Pennsylvania, Philadelphia; 10 Perelman School of Medicine, Department of Biostatistics, Epidemiology and Informatics, University of Pennsylvania, Philadelphia; 11 Department of Physical Therapy, University of Toronto, Toronto, Ontario, Canada; 12 Department of Physiology, University of Toronto, Toronto, Ontario, Canada

## Abstract

**Importance:**

Low diaphragm muscle mass at the outset of mechanical ventilation may predispose critically ill patients to poor clinical outcomes.

**Objective:**

To determine whether lower baseline diaphragm thickness (Tdi) is associated with delayed liberation from mechanical ventilation and complications of acute respiratory failure (reintubation, tracheostomy, prolonged ventilation >14 days, or death in the hospital).

**Design, Setting, and Participants:**

Secondary analysis (July 2018 to June 2019) of a prospective cohort study (data collected May 2013 to January 2016). Participants were 193 critically ill adult patients receiving invasive mechanical ventilation at 3 intensive care units in Toronto, Ontario, Canada.

**Exposures:**

Diaphragm thickness was measured by ultrasonography within 36 hours of intubation and then daily. Patients were classified as having low or high diaphragm muscle mass according to the median baseline Tdi.

**Main Outcomes and Measures:**

The primary outcome was time to liberation from ventilation accounting for the competing risk of death and adjusting for age, body mass index, severity of illness, sepsis, change in Tdi during ventilation, baseline comorbidity, and study center. Secondary outcomes included in-hospital death and complications of acute respiratory failure.

**Results:**

A total of 193 patients were available for analysis; the mean (SD) age was 60 (15) years, 73 (38%) were female, and the median (interquartile range) Sequential Organ Failure Assessment score was 10 (8-13). Median (interquartile range) baseline Tdi was 2.3 (2.0-2.7) mm. In the primary prespecified analysis, baseline Tdi of 2.3 mm or less was associated with delayed liberation from mechanical ventilation (adjusted hazard ratio for liberation, 0.51; 95% CI, 0.36-0.74). Lower baseline Tdi was associated a higher risk of complications of acute respiratory failure (adjusted odds ratio, 1.77; 95% CI, 1.20-2.61 per 0.5-mm decrement) and prolonged weaning (adjusted odds ratio, 2.30; 95% CI, 1.42-3.74). Lower baseline Tdi was also associated with a higher risk of in-hospital death (adjusted odds ratio, 1.47; 95% CI, 1.00-2.16 per 0.5-mm decrement), particularly after discharge from the intensive care unit (adjusted odds ratio, 2.68; 95% CI, 1.35-5.32 per 0.5-mm decrement).

**Conclusions and Relevance:**

In this study, low baseline diaphragm muscle mass in critically ill patients was associated with prolonged mechanical ventilation, complications of acute respiratory failure, and an increased risk of death in the hospital.

## Key Points

**Question:**

In mechanically ventilated patients, is diaphragm muscle mass at the outset of mechanical ventilation associated with clinical outcomes?

**Findings:**

In this cohort study of 193 critically ill adults receiving invasive mechanical ventilation, lower baseline diaphragm thickness was independently associated with a substantial delay in liberation from mechanical ventilation, prolonged weaning, and a higher risk of complications of acute respiratory failure. Lower baseline diaphragm thickness was also associated with higher in-hospital mortality, particularly after discharge from the intensive care unit.

**Meaning:**

This study found that low baseline diaphragm muscle mass was associated with prolonged mechanical ventilation and a higher risk of in-hospital death.

## Teaser

This cohort study examines whether baseline diaphragm muscle mass is associated with delayed liberation from mechanical ventilation and complications of acute respiratory failure in critically ill adults.

## Introduction

Skeletal muscle mass and function are important factors associated with outcomes in critically ill patients.^1,2,3,4,5,6,7,8,9^ While muscular atrophy and weakness are well-recognized consequences of critical illness,^10,11^ there is growing appreciation for the impact of premorbid health status and muscle function on the outcomes of critical illness. Baseline frailty is associated with a higher risk of death and long-term disability after critical illness,^12,13^ possibly because of diminished physiological reserve and reduced axial skeletal muscle mass and strength (sarcopenia).^2,3,4,5,14,15^

The association between premorbid diaphragm muscle mass and the outcomes of critical illness is unknown. Diaphragm muscle mass varies widely in the general population^16,17,18^; reduced diaphragm muscle mass has been documented in a variety of neuromuscular conditions.^19,20,21,22,23^ The diaphragm is the primary muscle of the respiratory pump, and reduced diaphragm muscle mass impairs inspiratory strength and cough function.^18,24,25,26^ In mechanically ventilated patients, diaphragm weakness is associated with increased risk of prolonged ventilation, intensive care unit (ICU) readmission, and death up to 1 year after ICU discharge.^27,28,29,30^ Consequently, low premorbid diaphragm muscle mass at the outset of critical illness may predispose patients to prolonged ventilator dependence and increase the risk of death or long-term functional disability. In this case, reduced diaphragm muscle mass and strength might offer a potential therapeutic target for patients in whom an episode of mechanical ventilation may be anticipated (eg, preoperative inspiratory muscle training before major surgery or transplantation).^31^

We hypothesized that low baseline diaphragm muscle mass at the outset of mechanical ventilation—as reflected by reduced thickness of the right hemidiaphragm—is associated with prolonged mechanical ventilation and poor clinical outcomes in mechanically ventilated patients. This hypothesis was tested in a secondary analysis of a previously reported prospective cohort study of diaphragm ultrasonography during mechanical ventilation.^11,32^ We also aimed to determine whether this association would be present in a subgroup of patients in whom the need for mechanical ventilation can be anticipated: patients requiring mechanical ventilation after solid-organ transplantation. Finally, we examined whether patients with greater diaphragm muscle mass at baseline would be at higher risk of ventilator-associated diaphragm atrophy.

## Methods

### Study Population and Setting

In this study, patients were drawn from 2 closely related cohort studies involving diaphragm ultrasonography in 3 intensive care units in Toronto, Ontario, Canada between May 2013 and January 2016: a large prospective study involving daily measurements of diaphragm thickness (Tdi) (cohort A), and a smaller prospective study involving daily measurements of Tdi together with continuous monitoring of diaphragm electrical activity by crural electromyography (cohort B) (eTable 1 in the Supplement). The analysis for the present study was conducted between July 2018 and June 2019.

Patients were eligible for enrollment in cohort A if they were invasively mechanically ventilated for less than 36 hours (initially, patients were eligible to be enrolled up to 72 hours after intubation; for the present analysis, patients were excluded if the first Tdi measurement was obtained >36 hours after intubation). Patients were excluded if they were expected to be liberated within 24 hours of screening or if they had received invasive ventilation for more than 48 hours in the previous 6 months. In cohort B, patients were eligible for inclusion if they were intubated for less than 36 hours because of acute brain injury, moderate or severe acute respiratory distress syndrome, septic shock, or pneumonia (all would have been eligible for enrollment in cohort A). Patients were excluded if they were deemed unlikely to remain on the ventilator for at least 7 days or if there were clinical conditions that interfered with reliable crural electromyographic measurements. Eligible patients were identified by regular screening (Monday to Thursday; screening was not performed over the weekend).

Informed consent was obtained from substitute decision makers before enrollment. In the absence of a substitute decision maker, patients were enrolled by deferred consent, and consent for the use of study data was obtained from study participants once they regained capacity. The research ethics boards at University Health Network and St Michael’s Hospital approved the study protocols. We followed the Strengthening the Reporting of Observational Studies in Epidemiology (STROBE) guideline for reporting cohort studies.^33^

### Measurement of Tdi and Definitions of Low Baseline Tdi and Diaphragm Atrophy

Using a validated technique,^34^ right Tdi was measured using a high-frequency (13 MHz) linear array transducer in the zone of apposition^35^ between the anterior and midaxillary lines at the level of the 9th or 10th intercostal space. Ultrasonographic probe placement location was marked to enhance day-to-day measurement consistency. End-expiratory Tdi was measured on 2 consecutive breaths from 2 separate images. Measurements were repeated at least once until consistently within 10%; the mean of all 4 measurements was used for analysis.^11,32,34^ Measurements were made daily on weekdays until extubation or day 14 of invasive mechanical ventilation.

Baseline Tdi was defined as the first Tdi measurement obtained by ultrasonography within 36 hours after the initiation of invasive mechanical ventilation.

### Patient Characteristics

Demographic data, comorbidities, admission diagnosis, and severity of illness (Simplified Acute Physiology Score II)^36^ were collected. Ventilator settings, arterial blood gas tensions, criteria for sepsis,^37^ Riker Sedation-Agitation Scale,^38^ exposure to neuromuscular blockade, and Sequential Organ Failure Assessment^39^ scores were recorded daily. Under the hypothesis that baseline Tdi would vary according to the severity of chronic cardiopulmonary disease, patient medical records were reviewed retrospectively for left ventricular ejection fraction, forced expiratory volume, and forced vital capacity measured within the 3 months prior to ICU admission.

### Clinical Outcomes

Patients were followed up until hospital discharge for the following events: extubation, reintubation, tracheostomy, ICU discharge, hospital discharge, and death. Liberation from ventilation was defined as separation from ventilation (extubation or tracheostomy mask breathing for 24 hours) without resumption of invasive ventilatory support during the index ICU admission. The ICUs where patients were enrolled shared a common standard sedation and weaning protocol (eAppendix in the Supplement). The primary end point was the time from intubation until liberation from ventilation (or death) over the first 21 days after intubation. Ventilator-free days were computed as the time from liberation from ventilation to day 60; patients who required more than 60 days of ventilatory support or who died on or before day 60 were assigned a value of 0. Complications of acute respiratory failure were defined as the occurrence of any of the following events: reintubation, tracheostomy, prolonged ventilation (>14 days), or death.^40,41^ Weaning duration was defined according to the previously published WIND (Weaning According to a New Definition) criteria.^42^ Investigators responsible for analysis of diaphragm ultrasonographic images were blinded to patient outcomes. Clinicians responsible for medical decisions, including weaning, were not aware of ultrasonographic measurement data.

### Statistical Analysis

In the primary analysis, the association between baseline Tdi and the rate of liberation from mechanical ventilation to day 21 was evaluated by nonparametric tests for differences in cumulative incidence and by competing risks regression using the method of Fine and Gray (eAppendix in the Supplement).^43^ The model was adjusted for prespecified covariates including age, sex, body mass index, Simplified Acute Physiology Score II, diagnosis of sepsis according to the Third International Consensus Definitions for Sepsis and Septic Shock,^37^ baseline Sequential Organ Failure Assessment score, baseline ratio of partial pressure of oxygen (Pao_2_) to fraction of inspired oxygen (Fio_2_), neuromuscular blockade during the first 48 hours of mechanical ventilation, baseline Sedation-Agitation Scale score, the presence of at least 1 chronic comorbidity, and center (eTable 2 in the Supplement). The initial change in Tdi during ventilation was also included in the model as a potential confounder after univariate analyses found that this variable (previously shown to be associated with outcome)^32^ was associated with baseline Tdi. The initial change in Tdi was defined as in our previous study: the change in Tdi on the first day that it exceeded a 10% increase or 10% decrease in thickness from baseline before day 7. Rates of missing data were low for all model covariates (eTable 3 in the Supplement). Baseline Tdi was dichotomized according to the median value based on the prespecified analysis plan. To address concerns about possible arbitrariness in this dichotomization, the same model was also computed treating baseline Tdi as a continuous variable.

Three additional sensitivity analyses were performed on the primary model: the analysis excluded the initial change in Tdi during mechanical ventilation; the analysis was restricted to patients where baseline Tdi was obtained within 18 hours of initiating mechanical ventilation; and the analysis was restricted to cohort A.

Additional multivariable linear, logistic, and Poisson regression models were constructed to assess the association between baseline Tdi and secondary outcomes (eAppendix in the Supplement). Potential associations between baseline Tdi and patient characteristics (age, sex, height, weight, body mass index, admission diagnoses, and comorbidities) were evaluated by linear regression. The association between baseline Tdi and the rate of change in Tdi over time during mechanical ventilation was assessed by linear mixed-effects regression.

A preplanned subgroup analysis was conducted in patients admitted to the ICU after solid-organ transplantation. This subpopulation was of interest because the expectation of transplantation entails that the requirement for mechanical ventilation can be predicted; baseline Tdi therefore represents a potentially modifiable exposure in these patients.

Statistical significance was set at 2-sided *P* < .05. All statistical analyses were conducted using R software version 3.3.2 (R Project for Statistical Computing).

## Results

### Study Cohort

A total of 224 patients were prospectively enrolled. Consent for participation was withdrawn for 7 patients, 1 patient died before baseline Tdi was measured, 2 patients were lost to follow-up because of transfer to other hospitals prior to liberation from mechanical ventilation, and baseline Tdi was not obtained within 36 hours of intubation for 21 patients, leaving 193 patients for analysis (eFigure 1 in the Supplement). The mean (SD) age was 60 (15) years, 73 patients (38%) were female, and the median (interquartile range [IQR]) Sequential Organ Failure Assessment score was 10 (8-13) (eTable 4 in the Supplement). Admission diagnoses are detailed in eTable 5 in the Supplement. A total of 44 patients (23%) died in the ICU, 65 (34%) died in the hospital (ICU and post-ICU), and 105 (55%) experienced at least 1 complication of acute respiratory failure (Table).

**Table.** Baseline Tdi and Clinical Outcomes

Outcome	No. (%)			Unadjusted *P* Value	Adjusted Association per 0.5-mm Decrement in Tdi (95% CI)^b^
	Total (N = 193)	Tdi ≤2.3 mm (n = 105)^a^	Tdi >2.3 mm (n = 88)		
Competing risk analysis to day 21					
Liberation from ventilation	130 (67)	65 (62)	65 (74)	.04	Hazard ratio, 0.84 (0.70-1.00)
Death while receiving ventilation	30 (16)	15 (14)	15 (17)	.52	Hazard ratio, 0.62 (0.34-1.13)
Hospital mortality	65 (34)	44 (43)	21 (24)	.01	Odds ratio, 1.47 (1.00-2.16)
Death in ICU	44 (23)	27 (26)	17 (19)	.38	Odds ratio, 1.03 (0.67-1.57)
Death in hospital in ICU survivors	21 (14)	17 (22)	4 (6)	.01	Odds ratio, 2.68 (1.35-5.32)
Ventilator-free days to day 60, median (IQR)	48 (0-55)	42 (0-54)	53 (36-56)	.001	Duration ratio, 0.89 (0.80-0.99)
Duration of mechanical ventilation in ICU survivors, median (IQR), d	7 (4-13)	8 (4-16)	6 (3-12)	.06	Duration ratio, 1.29 (1.13-1.47)
Duration of ICU admission in ICU survivors, median (IQR), d	9 (5-16)	10 (5-18)	8 (5-14)	.12	Duration ratio, 1.30 (1.14-1.48)
Duration of hospitalization in hospital survivors, median (IQR), d	24 (14-58)	28 (16-62)	22 (13-51)	.47	Duration ratio, 1.23 (1.04-1.46)
Complications of acute respiratory failure					
Reintubation	33 (17)	22 (21)	11 (12)	.17	Odds ratio, 1.91 (1.18-3.11)
Tracheostomy	34 (18)	22 (21)	12 (14)	.26	Odds ratio, 1.60 (1.01-2.53)
Mechanical ventilation >14 d	57 (30)	41 (39)	16 (18)	.003	Odds ratio, 1.80 (1.23-2.64)
Rate of ≥1 complication of mechanical ventilation^c^	105 (55)	67 (65)	38 (43)	.004	Odds ratio, 1.77 (1.20-2.61)
Readmission to ICU after ICU discharge	20 (11)	13 (13)	7 (9)	.45	Odds ratio, 1.37 (0.79-2.39)
Weaning classification^d^					
No attempt	32 (17)	18 (17)	14 (16)	.007	
Simple	75 (39)	34 (33)	41 (47)		
Difficult	45 (23)	21 (20)	24 (27)		
Prolonged	40 (21)	31 (30)	9 (10)		Odds ratio, 2.30 (1.42-3.74)^e^

Abbreviations: ICU, intensive care unit; IQR, interquartile range; Tdi, diaphragm thickness.

^a^ The 50th percentile of baseline Tdi was 2.3 mm (range, 1.0-4.2 mm).

^b^ Adjusted for age, body mass index, Simplified Acute Physiology II score, sepsis, baseline Sequential Organ Failure Assessment score, baseline partial pressure of arterial oxygen to fraction of inspired oxygen ratio, baseline Sedation-Agitation Scale score, use of neuromuscular blockade, presence of comorbidity, and center.

^c^ Composite of death in hospital, reintubation, tracheostomy, or prolonged mechanical ventilation more than 14 days.

^d^ Duration of weaning classified according to number of days required for liberation from mechanical ventilation after meeting readiness-to-wean criteria.^42^

^e^ Odds ratio for risk of prolonged weaning vs other weaning outcomes combined.

### Factors Associated With Baseline Tdi

Median (IQR) baseline Tdi was 2.3 (2.0-2.7) mm (range, 1.0-4.2 mm). Median (IQR) time from intubation to first ultrasonographic measurement was 21 (14-26) hours; there was no association between baseline Tdi and the time from intubation to first measurement (*R*^2^ < 0.01; *P* = .29). There was no difference in baseline Tdi between men (median [IQR], 2.3 [2.1-2.7] mm) and women (median [IQR], 2.3 [1.9-2.6] mm) (*P* = .43). Baseline Tdi was lower in cohort B compared with cohort A (median [IQR], 1.8 [1.6-2.2] mm vs 2.3 [2.1-2.7] mm; *P* = .004). Baseline Tdi was weakly correlated with patient weight and body mass index (eFigure 2 in the Supplement) (*R*^2^ = 0.02 and 0.03, respectively). Baseline Tdi was not associated with age, location prior to hospitalization, Charlson Comorbidity Index score, cause of respiratory failure, history of chronic obstructive lung disease, history of restrictive lung disease, history of congestive heart failure, left ventricular ejection fraction, or forced expiratory volume and forced vital capacity (eTable 4 in the Supplement).

### Baseline Tdi and Clinical Outcomes of Acute Respiratory Failure

**Figure 1.**
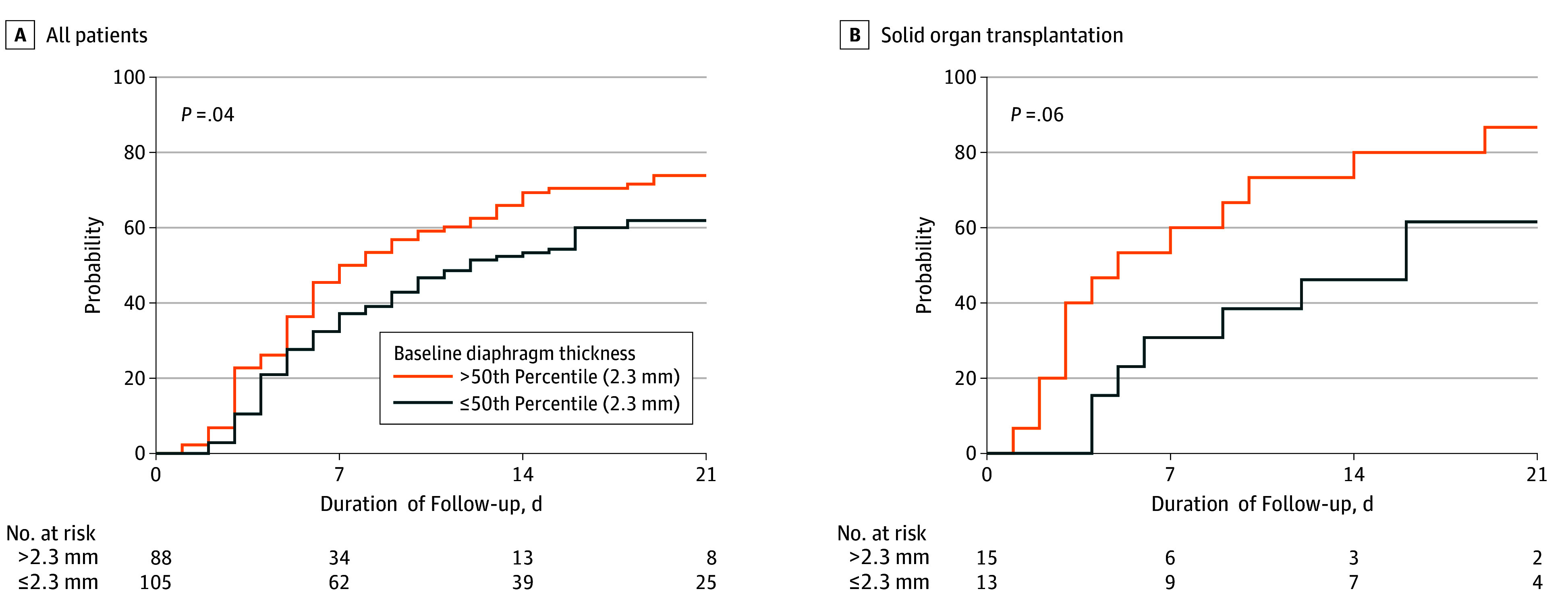
Baseline Diaphragm Thickness and the Cumulative Incidence of Liberation From Mechanical Ventilation to Day 21, Accounting for the Competing Risk of Death on Mechanical Ventilation A, At day 21, the cumulative incidence of liberation was lower in patients with baseline diaphragm thickness of 2.3 mm or less. B, In the subset of patients admitted to the intensive care unit for mechanical ventilation after solid-organ transplantation, the cumulative incidence of liberation from ventilation to day 21 was lower with baseline diaphragm thickness of 2.3 mm or less.

The cumulative incidence of liberation from mechanical ventilation was lower in patients with baseline Tdi at or below the 50th percentile (*P* = .04) (Figure 1A). In the primary prespecified analysis, baseline Tdi at or below the 50th percentile was associated with delayed liberation from ventilation (adjusted hazard ratio [HR], 0.51; 95% CI, 0.36-0.74) (Figure 1A). Similar associations were observed when expressing baseline Tdi as a continuous variable (adjusted HR, 0.84; 95% CI, 0.70-1.00 per 0.5-mm decrement) and when restricting the model to the 84 patients in whom baseline Tdi was measured within 18 hours of intubation (adjusted HR, 0.57; 95% CI, 0.40-0.81). Excluding change in Tdi from the model attenuated the association between baseline Tdi and liberation from mechanical ventilation (adjusted HR, 0.72; 95% CI, 0.52-1.02). The association was unchanged after restricting the analysis to cohort A (adjusted HR, 0.52; 95% CI, 0.36-0.76).

**Figure 2.**
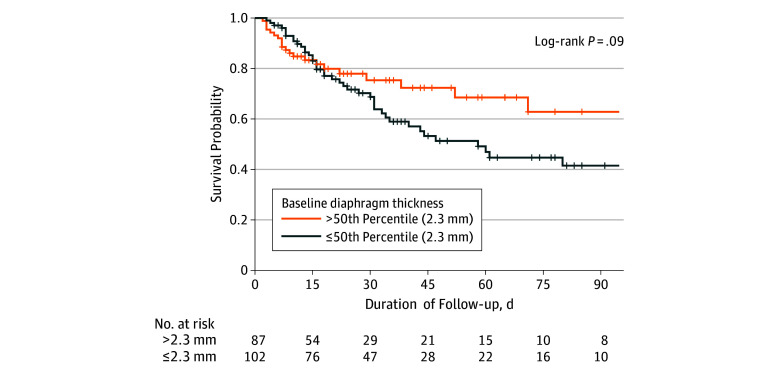
Baseline Diaphragm Thickness and Mortality Patients were censored at discharge from the hospital. Lower baseline diaphragm thickness was associated with higher hospital mortality.

**Figure 3.**
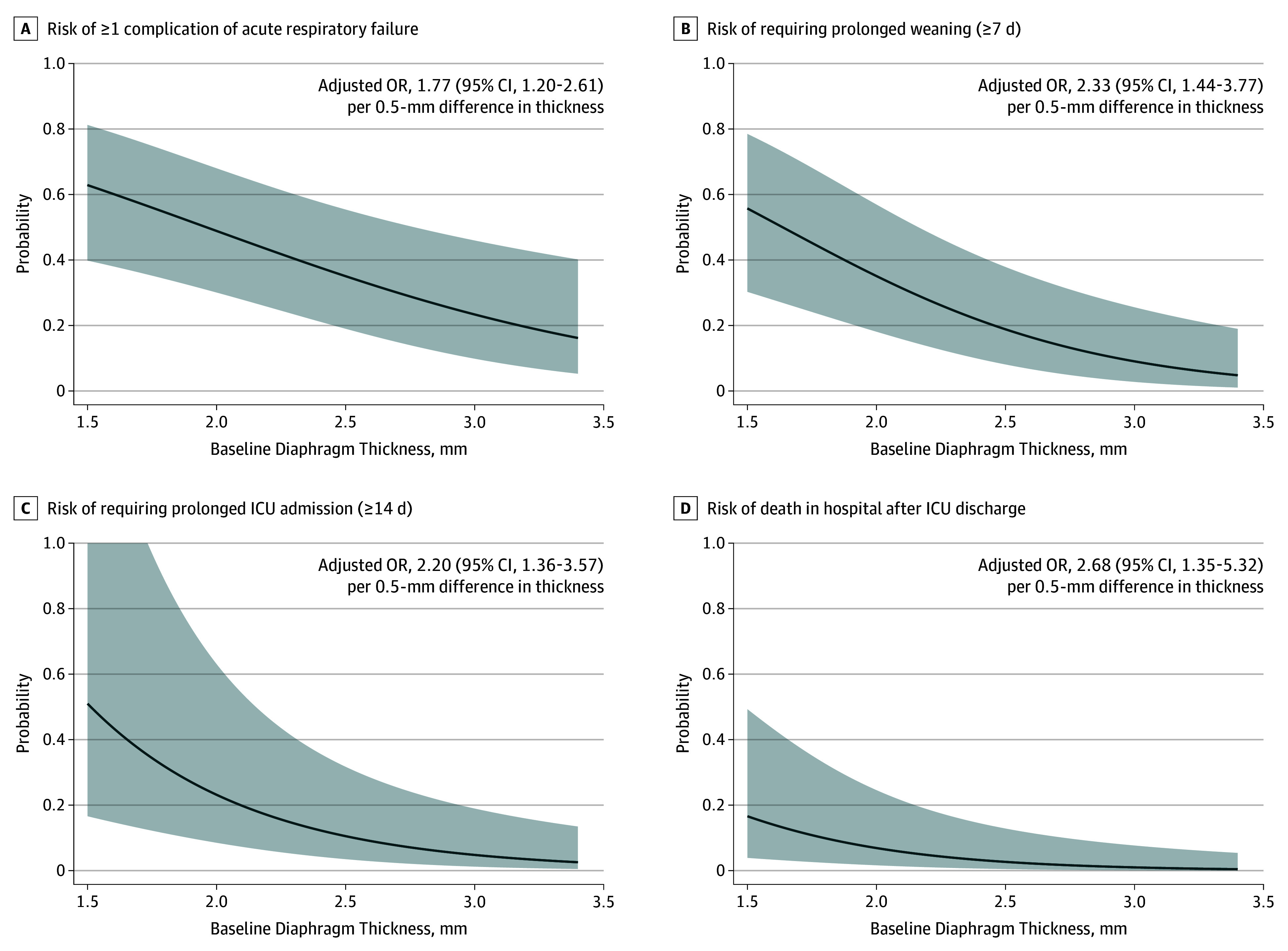
Baseline Diaphragm Thickness and Clinical Outcomes of Mechanical Ventilation Probabilities were adjusted for age, sex, body mass index, initial change in diaphragm thickness during ventilation, Simplified Acute Physiology Score II, sepsis, baseline Sequential Organ Failure Assessment score, baseline ratio of partial pressure of oxygen (Pao_2_) to fraction of inspired oxygen (Fio_2_), baseline Riker Sedation-Agitation Scale score, use of neuromuscular blockade, presence of comorbidity, and center. Shaded area indicates 95% CI; ICU, intensive care unit; and OR, odds ratio.

Lower baseline Tdi was also associated with significantly greater hospital mortality (adjusted odds ratio [OR], 1.47; 95% CI, 1.00-2.16 per 0.5-mm decrement) (Table and Figure 2), particularly after ICU discharge (adjusted OR, 2.68; 95% CI, 1.35-5.32 per 0.5-mm decrement) (Figure 3). Lower baseline Tdi was associated a higher risk of complications of acute respiratory failure (adjusted OR, 1.77; 95% CI, 1.20-2.61 per 0.5-mm decrement) and prolonged weaning (adjusted OR, 2.30; 95% CI, 1.42-3.74) (Table and Figure 3).

### Baseline Tdi and ICU Outcomes After Solid-Organ Transplantation

A total of 28 patients requiring invasive mechanical ventilation following solid-organ transplantation were enrolled (19 with lung transplantation, 18 with liver transplantation, and 1 with cardiac transplantation). In this subgroup, lower baseline Tdi was associated with prolonged mechanical ventilation (median [IQR], 16 [6-23] vs 5 [3-12] days; *P* = .03) (Figure 1B). The differences in duration of ICU stay (median [IQR], 16 [9-22] vs 7 [4-14] days; *P* = .06) and rate of complications of acute respiratory failure (69% vs 33%, *P* = .13) did not reach statistical significance.

### Baseline Tdi, Change in Tdi Over Time, and Outcome

**Figure 4.**
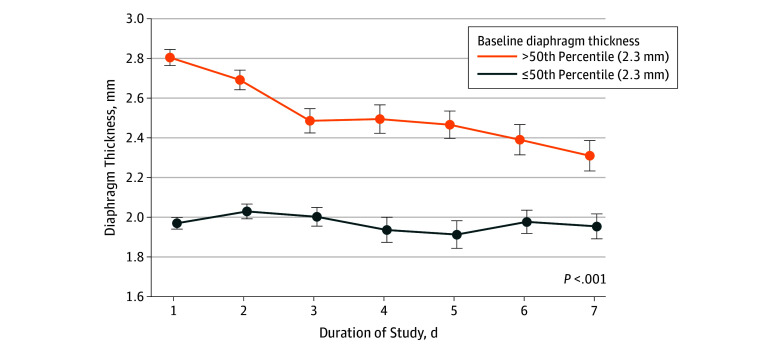
Change in Diaphragm Thickness From Baseline The change in mean diaphragm thickness over time during mechanical ventilation varied according to baseline diaphragm thickness. Error bars indicate standard error of the mean.

The rate of change in Tdi during mechanical ventilation varied significantly with baseline Tdi: patients with higher baseline Tdi exhibited a progressive decrease in mean Tdi, whereas mean Tdi was stable over time in patients with lower baseline Tdi (Figure 4; *P* < .001 for interaction). The association between change in Tdi during ventilation and clinical outcome did not differ significantly with lower and higher baseline Tdi (eFigure 3 in the Supplement) (*P* > .10 for interaction).

## Discussion

The central finding of this study is that reduced Tdi at the outset of mechanical ventilation was independently associated with prolonged mechanical ventilation, prolonged ICU admission, and a substantially greater risk of in-hospital death. Much of this excess risk of death was observed after ICU discharge independent of the initial severity of illness. We also found that higher baseline Tdi was associated with a substantially higher risk of developing diaphragm atrophy during mechanical ventilation.

Because of the unique geometry of the diaphragm, its thickness is linearly correlated with its cross-sectional area^18^ and, hence, to its muscle mass.^44^ Accordingly, we infer that lower Tdi reflects lower diaphragm muscle mass. The distribution of baseline Tdi reported in this study corresponds with values reported in other studies in healthy individuals^16,18^ and mechanically ventilated patients.^45,46,47^

The observed association between Tdi and clinical outcomes reported might arise by 2 different mechanisms. First, patients with low baseline Tdi may have globally reduced muscle mass^44^ accompanied by increased frailty and reduced global physiological reserve, factors that influence outcomes in critically ill patients.^12,13,14^ Consistent with this hypothesis, we found that weight and body mass index were weakly correlated with baseline Tdi, as shown in previous studies.^18,21^ However, other markers of chronic health status likely associated with globally reduced muscle mass and frailty, including age, chronic cardiopulmonary disease, comorbidity scores, and duration of hospitalization prior to ICU admission, were not associated with baseline Tdi. Of note, Vivier et al^48^ recently reported that Tdi was not correlated with pectoralis muscle thickness at the outset of mechanical ventilation, suggesting that baseline Tdi may not reflect global axial skeletal muscle mass.

Alternatively, the observed association may arise from a direct causal effect of reduced diaphragm muscle mass leading to impaired diaphragm function and inability of the patient to be separated from the ventilator. Decreases in muscle thickness and cross-sectional area reduce muscular force-generating capacity.^24^ Diaphragm strength (maximal inspiratory and transdiaphragmatic pressures) is correlated with Tdi in outpatients,^19,25^ and Tdi increases in parallel with increases in diaphragm strength following muscle training regimens^49^ or during recovery from diaphragm paralysis.^19^ In one study of critically ill patients, higher Tdi at the outset of mechanical ventilation was associated with greater diaphragm force-generating capacity.^21^ Diaphragm function is a crucial determinant of liberation from mechanical ventilation,^28^ and patients with lower baseline Tdi would be expected to have difficulties with liberation from mechanical ventilation. While a direct causal effect is plausible, this study cannot confirm this hypothesis. Of note, the increased risk of mortality associated with low baseline Tdi was predominantly due to death in the hospital after ICU discharge, suggesting that these deaths may be related to the sequelae of prolonged critical illness and/or baseline frailty rather than the initial severity of illness.

These findings have several important implications for clinical practice and research. First, baseline Tdi is independently associated with the risk of prolonged ICU admission and poor post-ICU outcomes. It may therefore prove to be a useful means for stratifying patients for early interventions to prevent and treat the complications of prolonged critical illness.

Second, strategies aimed at enhancing or preserving diaphragm muscle mass might improve clinical outcomes in patients at high risk of respiratory failure. This is particularly important in patients in whom an episode of mechanical ventilation may be anticipated, such as patients awaiting solid-organ transplantation, in whom there was an association between baseline Tdi and duration of ventilation. Inspiratory muscle training has been shown to improve exercise performance, inspiratory muscle strength, and quality of life among several populations with chronic cardiopulmonary dysfunction,^50,51,52,53^ and professional society guidelines recommend this therapy prior to major surgery.^54^ The benefit of inspiratory muscle training in patients awaiting solid-organ transplantation requires prospective confirmation in clinical trials.

Third, baseline Tdi can be used to stratify patients for the risk of diaphragm atrophy during mechanical ventilation. Decreases in Tdi during mechanical ventilation consistent with the well-described phenomenon of ventilator myotrauma^55^ were substantially greater and more frequent in patients with higher baseline thickness compared with patients with lower baseline thickness.^11,32^ The mechanism of this finding is unclear. There may be a floor effect for diaphragm atrophy as there is for other muscles,^56,57^ and patients presenting with reduced Tdi at baseline may therefore have limited potential for atrophy. Of note, the magnitude of disuse atrophy during bed rest was shown to correlate with baseline muscular cross-sectional area in some muscles of the lower extremities.^58^ While the association between baseline Tdi and change in Tdi might theoretically arise from regression to the mean, such a purely statistical phenomenon would not account for the strong association between changes in Tdi and clinical outcomes. Indeed, the association between changes in Tdi during ventilation and clinical outcomes was substantially greater in patients with higher baseline Tdi. These findings suggest that diaphragm-protective ventilation strategies^59^ may exert the greatest clinical benefit in patients with higher baseline Tdi.

It may seem counterintuitive that higher baseline Tdi would be associated with both better clinical outcomes and an increased risk of developing ventilator-associated diaphragm atrophy (which is, in turn, associated with worse clinical outcomes). Such 3-variable associations are well recognized in epidemiology: in this case, the development of atrophy functions as a so-called suppressor variable (in contrast to a mediator variable), such that including it in the regression equation strengthens the association between baseline Tdi and outcome.^60^ For this reason, the association between baseline Tdi and the time to liberation from mechanical ventilation was stronger after adjusting for the development of changes in Tdi from baseline. The decision to include changes in Tdi in the regression model reflected our specific interest in the mechanistic question of the extent to which baseline Tdi per se may influence outcomes, as opposed to the purely prognostic question of the extent to which baseline Tdi predicts outcomes. The association between baseline Tdi and outcome was similar, although slightly attenuated, in the sensitivity analysis excluding change in Tdi from the model.

### Limitations

This study has several limitations. First, we did not measure diaphragm strength in this cohort. This limits our ability to establish the mechanism by which baseline Tdi was associated with outcome. Second, we do not have measures of frailty prior to ICU admission. Given the link between frailty and sarcopenia,^14^ the association between preadmission frailty and baseline Tdi should be more extensively characterized beyond Charlson Comorbidity Index score and pre-ICU cardiopulmonary comorbidities.

Third, baseline Tdi measurements were made up to 36 hours from the onset of mechanical ventilation. During this period, Tdi may be affected by mechanical ventilation, sepsis, and other physiological derangements, raising questions as to the validity of the initial Tdi measurement as a true baseline measurement. Ideally, patients would have Tdi measurements done as early as possible in their course of critical illness. Reassuringly, there was no correlation between time from intubation to first measurement and baseline Tdi, and the association between baseline Tdi and outcome persisted in a sensitivity analysis restricted to patients in whom Tdi was measured within 18 hours of intubation. Moreover, the time from intubation to baseline measurement was similar between patients with higher or lower baseline Tdi.

Fourth, this study is a secondary analysis of a previously published observational study.^32^ While the analysis plan was specified a priori on the basis of plausible biological rationale for an association between baseline Tdi and prolonged ventilation, future studies should aim to replicate our findings and to establish whether modifying Tdi can improve clinical outcome.

## Conclusions

In this study, reduced baseline Tdi was associated with prolonged mechanical ventilation, an increased risk of complications of acute respiratory failure, and increased in-hospital mortality. Higher baseline Tdi was associated with a higher risk of developing diaphragm atrophy during mechanical ventilation.

## Notes

Supplement. **eTable 1.** Design Characteristics of the Two Cohort Studies Enrolling Patients for This Study **eAppendix. **Additional Methods **eTable 2.** Exposure Variable Selection for All Multivariable Models **eTable 3.** Frequency of Missing Variables in Clinical Outcomes **eTable 4.** Patient Characteristics According to Baseline Diaphragm Thickness **eTable 5.** Admission Categories and Diagnoses **eFigure 1.** CONSORT Diagram and Flow of Study Patients **eFigure 2.** Right Hemidiaphragm Thickness Measured at the Outset of Mechanical Ventilation Was Weakly Correlated With Patient Height, Weight, and Body Mass Index **eFigure 3.** The Influence of Baseline Diaphragm Thickness on the Association Between Change in Diaphragm Thickness During Mechanical Ventilation and Clinical Outcomes **eReferences**.
