# Supplementary material for: Association of Low Baseline Diaphragm Muscle Mass With Prolonged Mechanical Ventilation and Mortality Among Critically Ill Adults
Source: JAMA Netw Open. 2020 Feb 19;3(2):e1921520. doi: 10.1001/jamanetworkopen.2019.21520 (PMC12124489; doi:10.1001/jamanetworkopen.2019.21520)
Supplement: Supplement. — eTable 1. Design Characteristics of the Two Cohort Studies Enrolling Patients for This Study eAppendix. Additional Methods eTable 2. Exposure Variable Selection for All Multivariable Models eTable 3. Frequency of Missing Variables in Clinical Outcomes eTable 4. Patient Characteristics According to Baseline Diaphragm Thickness eTable 5. Admission Categories and Diagnoses eFigure 1. CONSORT Diagram and Flow of Study Patients eFigure 2. Right Hemidiaphragm Thickness Measured at the Outset of Mechanical Ventilation Was Weakly Correlated With Patient Height, Weight, and Body Mass Index eFigure 3. The Influence of Baseline Diaphragm Thickness on the Association Between Change in Diaphragm Thickness During Mechanical Ventilation and Clinical Outcomes eReferences. [file jamanetwopen-e1921520-s001.pdf]

Supplementary Online Content

Sklar MC, Dres M, Fan E, et al. Association of low baseline diaphragm muscle mass with prolonged mechanical ventilation and mortality among critically ill adults. *JAMA Netw Open*. 2020;3(2):e1921520. doi:10.1001/jamanetworkopen.2019.21520

eTable 1. Design Characteristics of the Two Cohort Studies Enrolling Patients for This Study
eAppendix. Additional Methods
eTable 2. Exposure Variable Selection for All Multivariable Models
eTable 3. Frequency of Missing Variables in Clinical Outcomes
eTable 4. Patient Characteristics According to Baseline Diaphragm Thickness
eTable 5. Admission Categories and Diagnoses
eFigure 1. CONSORT Diagram and Flow of Study Patients
eFigure 2. Right Hemidiaphragm Thickness Measured at the Outset of Mechanical Ventilation Was Weakly Correlated With Patient Height, Weight, and Body Mass Index
eFigure 3. The Influence of Baseline Diaphragm Thickness on the Association Between Change in Diaphragm Thickness During Mechanical Ventilation and Clinical Outcomes
eReferences.

This supplementary material has been provided by the authors to give readers additional information about their work.

© 2020 Sklar MC et al. *JAMA Network Open.*

eTable 1

Design characteristics of the two cohort studies enrolling patients for this study

**Study Parameter**	**Cohort A**	**Cohort B**
Objective	• To describe the changes in diaphragm thickness over time during mechanical ventilation<br>• To ascertain the influence of mechanical ventilation settings and inspiratory effort under mechanical ventilation on diaphragm thickness<br>• To examine the relationship between diaphragm thickness and clinical outcomes	• To ascertain the relationship between diaphragm inactivity and changes in diaphragm thickness over the early course of mechanical ventilation<br>• To precisely quantify the variation in diaphragm activity within and between patients during the early course of mechanical ventilation<br>• To examine the relationship between diaphragm thickness and clinical outcomes
Inclusion Criteria	Patients receiving invasive ventilation for less than 36 hours* for acute respiratory failure of any cause.	Patients receiving invasive ventilation for less than 36 hours** for:<br>• Acute severe brain injury (GCS < 8 prior to intubation)<br>• Moderate or severe acute respiratory distress syndrome (P/F < 200 mm Hg)<br>• Septic shock (proven or suspected infection, 2 SIRS criteria satisfied, patient requiring vasopressor support)<br>• Pneumonia (clinically suspected or confirmed)
Exclusion Criteria	• Anticipated duration of ventilation from time of screening is less than 24 hours<br>• Mechanical ventilation for more than 48 hours in the preceding 6 months	• Predicted probability of remaining alive and on the ventilator on ICU day 7 is less than 50% (PI judgment based on clinical data)<br>• Liberation from MV anticipated within 24 hours of screening<br>• High cervical spine injury<br>• Previously diagnosed neuromuscular disease<br>• Acute exacerbation of obstructive lung disease<br>• Known esophageal varices or esophageal injury or recent upper GI tract surgical procedures<br>• Mechanical ventilation for more than 48 hours in the preceding 6 months
Number of centers participating in study	Multi-center (3 ICUs at 2 institutions)	Single-center (2 ICUs at 1 institution)

© 2020 Sklar MC et al. *JAMA Network Open.*

Time period of patient enrolment	May 2013 to January 2016	January 2014 to March 2016
Ultrasound measurements	Diaphragm thickness and thickening fraction were measured once daily for the first 14 days of mechanical ventilation (or until death or extubation, if earlier)	Diaphragm thickness and thickening fraction were measured once daily for the first 14 days of mechanical ventilation (or until death or extubation, if earlier)
Clinical measurements	• Demographic and clinical characteristics<br>• Critical illness characteristics (cause, baseline severity, evolution of illness)	• Demographic and clinical characteristics<br>• Critical illness characteristics (cause, baseline severity, evolution of illness)
Diaphragm electrical activity measurements	n/a	Recorded on an hourly basis from time of enrolment until completion of 7 days of mechanical ventilation (or liberation or death, if prior to 7 days of MV)
Clinical outcome measurements	• Duration of mechanical ventilation<br>• Survival status at hospital discharge<br>• Ventilator-free days to day 60<br>• Reintubation<br>• Tracheostomy	• Duration of mechanical ventilation<br>• Survival status at hospital discharge<br>• Ventilator-free days to day 60<br>• Reintubation<br>• Tracheostomy

*Initial duration of ventilation stipulated was 72 hours; this was modified to minimize time from intubation to first measurement after the first 53 subjects were enrolled after observing that most subjects could be enrolled within 36 hours.

**Initial duration of ventilation prior to enrolment was 12 hours; this was modified to 36 hours after observing that patients could not feasibly enrolled within that time frame.

© 2020 Sklar MC et al. *JAMA Network Open.*

eAppendix: Additional Methods

Sedation and weaning practices in participating study intensive care units

The intensive care units that participated in this study use a harmonized sedation and weaning strategy. Sedative infusions are generally titrated to achieve a Riker Sedation-Agitation Scale[1] of 3-4 and the transition from controlled modes to spontaneous modes of mechanical ventilation as early as possible (depending on severity of patient illness). Choice of sedative infusion is left to the discretion of the clinician but local culture favours propofol and opioid infusions over benzodiazepines (depending on clinical scenarios). The use of dexmedetomidine is reserved for patients with agitation despite optimization of oral and intravenous adjuncts and at the time of weaning from mechanical ventilation. The use of neuromuscular blockade is typically reserved for cases of severe hypoxemic respiratory failure and refractory intracranial hypertension. In cases where neuromuscular blockade is required sedation targets are intensified to a SAS of 1-2, to ensure adequate sedation while paralysis is utilized. The timing of tracheostomy is not protocolized and is left to the discretion of the medical team.

Aggressive early mobility and physiotherapy are standard for all mechanically ventilated patients. Sedated patients will undergo passive range of motion exercises and awake ventilated patients will be mobilized as tolerated according to clinical scenario.

An interruption of continuous sedative infusions occurs daily (in patients not receiving neuromuscular blockers) and patients are screened by respiratory therapists for safety in administering a trial of spontaneous breathing once per day. In brief, patients on assisted modes of mechanical ventilation with inspired oxygen requirements ≤ 50% and positive end-expiratory pressure ≤ 10 cm H_20 as well as minimal vasopressor support, no evidence of myocardial ischemia, elevation of intracranial pressure and absence of agitation will undergo a trial of spontaneous breathing. In the participating ICUs, a spontaneous breathing trial is conducted on 0 cm H_20 of pressure support and 0 cm H_20 of positive end-expiratory pressure for 60 minutes. Patients are monitored for success or failure based on clinical criteria, with failure defined as a rapid shallow breathing index of > 105 breaths/minute/litre, use of accessory muscles of respiration, abdominal paradox, diaphoresis, hypertension and/or tachycardia and desaturation events. As part of a weaning and readiness for extubation protocol, patients are also examined for neurological status, cough strength and secretion burden. The decision to extubate is left to the medical team as is the modality of oxygen therapy (ie. high-flow nasal cannula, non-invasive ventilation of conventional face mask) post extubation.

© 2020 Sklar MC et al. *JAMA Network Open.*

Model Building Procedures

Primary model: Fine-Gray competing risks regression on time to disconnection from mechanical ventilation

Patients can be disconnected from the ventilator for one of two reasons: recovery and liberation from the ventilator, or death. Because a given exposure has the potential to modify either or both these competing events, modelling time to liberation from mechanical ventilation must account for the problem of competing risks. Following the proposal of Yehya et al.,[2] we employed the Fine-Gray competing risks regression.

The primary outcome of interest was the rate of liberation from mechanical ventilation over the first 21 days. Twenty-one days was selected as the time frame because day 21 is an established definition for chronic critical illness and prolonged mechanical ventilation[3] and accorded with our analysis in a previous paper on the association between changes in diaphragm thickness during mechanical ventilation and delayed liberation from mechanical ventilation.[4]

Liberation from mechanical ventilation was defined as disconnection from the ventilator alive without requiring reinstitution of ventilatory support during the index ICU admission. The competing event of disconnection because of death on mechanical ventilation was modelled.

The primary exposure variable of interest was baseline diaphragm thickness. This exposure was modelled using both continuous and dichotomous approaches (baseline diaphragm thickness was dichotomized at the median value); a dichotomous approach was employed per the pre-specified analysis plan.

The model was adjusted for all covariates listed in **Supplemental Table 3**. The initial change in diaphragm thickness was modelled as a non-linear term using restricted cubic splines (3 knots) given the non-linear relationship we identified in a previous publication. The rationale for inclusion of each covariate is given in **Supplemental Table 3**. Given that this model is designed to assess the independent relation between baseline diaphragm thickness and outcome, no methods were used to enhance model parsimony.

Sensitivity analyses on the model included:

1. Modelling baseline Tdi as a continuous variable
2. Excluding initial change in Tdi as a covariate
3. Restricting to patients in whom measurements were obtained within 18 hours of intubation (instead of 36 hours)
4. Restricting to patients enrolled in Cohort A

Modelling dichotomous end points

A series of binary second-endpoints were modelled, including hospital mortality, ICU mortality, reintubation, tracheostomy, mechanical ventilation > 14 days, readmission, prolonged weaning (> 7 days), or development of at least one complication of respiratory failure (a composite of reintubation, tracheostomy, prolonged mechanical ventilation > 14 days, or death in hospital).

© 2020 Sklar MC et al. *JAMA Network Open.*

Multivariable logistic regression models were constructed for each of these binary endpoints. Baseline diaphragm thickness was modelled as a continuous exposure and the initial change in diaphragm thickness was modelled using restricted cubic splines to account for non-linearity. All covariates listed in **Supplemental Table 3** were incorporated in the model.

Modelling continuous measures of duration of time

Duration of mechanical ventilation, duration of ICU admission, and duration of hospitalization were modelled using multivariable linear regression. To account for the competing risk of death, the first two models were restricted to ICU survivors, and the third model was restricted to hospital survivors. The outcomes were modelled using the logarithm of each endpoint to obtain a normal distribution for linear modelling. Accordingly, the coefficients obtained from the model provide the logarithm of the duration ratio for a given difference in baseline diaphragm thickness. Baseline diaphragm thickness was modelled as a continuous exposure and the initial change in diaphragm thickness was modelled using restricted cubic splines to account for non-linearity. All covariates listed in **Supplemental Table 3** were incorporated in the model.

To model ventilator-free days to day 60, a generalized linear model was fit using a quasipoisson distribution to account for overdispersion in the distribution of ventilator-free days. The coefficients obtained from the model provide the logarithm of the count ratio (effectively a duration ratio) for a given difference in baseline diaphragm thickness. Baseline diaphragm thickness was modelled as a continuous exposure and the initial change in diaphragm thickness was modelled using restricted cubic splines to account for non-linearity. All covariates listed in **Supplemental Table 3** were incorporated in the model.

Modelling the association between baseline diaphragm thickness and the rate of change in diaphragm thickness

To quantify the association between baseline diaphragm thickness and the rate of change in diaphragm thickness over time, we constructed a linear mixed-effects regression model of the interactive effects of time and baseline diaphragm thickness on daily diaphragm thickness measurements with a random effects term to account for repeated measures within subjects. Because daily diaphragm thickness exhibited a skewed distribution, the model was constructed using the logarithm of diaphragm thickness as the outcome.

Power calculation

The available study sample (n=193) gave 85% power to detect a hazard ratio for liberation of 0.60 (equivalent to a 10% difference in the cumulative incidence of liberation at day 21) at a significance level of 0.05, assuming a hazard ratio for the competing effect of death on mechanical ventilation of 1.0, a cumulative incidence of liberation at day 21 of 70%, and a cumulative incidence of death on mechanical ventilation at day 21 of 20%.

© 2020 Sklar MC et al. *JAMA Network Open.*

eTable 2

Exposure variable selection for all multivariable models

Exposure variable	Rationale for inclusion	Missing frequency (%)
Baseline diaphragm thickness (mm)	Primary exposure variable	0/193 (0%)
Initial change in diaphragm thickness (% of baseline diaphragm thickness)	Previously shown to be associated with outcome[4]	0/193 (0%)
Age	Potential determinant of outcome	0/193 (0%)
Body mass index	Potential determinant of outcome and potentially associated with diaphragm thickness	3/193 (1.5%)
Sex	Potential determinant of outcome and potentially associated with diaphragm weakness	0/193 (0%)
SAPS II score	Measure of illness severity, used to adjust for potential confounding by illness severity	0/193 (0%)
SOFA score	Measure of organ failure, used to adjust for potential confounding by illness severity	0/193 (0%)
PaO_2/FiO_2 ratio	Measure of severity of hypoxemia, used to adjust for potential confounding by illness severity	0/193 (0%)
Diagnosis of sepsis at baseline (SEPSIS-III criteria)	Presence of sepsis is known to be associated with both abnormal diaphragm dysfunction and a higher risk of death and prolonged mechanical ventilation	0/193 (0%)
Presence of at least one comorbidity	Comorbidity is an important potential confounder between diaphragm thickness and clinical outcome	0/193 (0%)
Riker Sedation-Agitation Scale at baseline	Sedation depth is strongly associated with the risk of prolonged mechanical ventilation	0/193 (0%)
Neuromuscular blockade within 48 hours of admission	Use of neuromuscular blockade may be associated with the risk of prolonged mechanical	0/193 (0%)

© 2020 Sklar MC et al. *JAMA Network Open.*

	ventilation and possibly with diaphragm dysfunction	
Center	Included in the model to account for possible center effects on clinical outcomes	0/193 (0%)

© 2020 Sklar MC et al. *JAMA Network Open.*

eTable 3
Frequency of missing variables in clinical outcomes

**Outcome variable**	**Missing frequency (%)**
Time to disconnection from mechanical ventilation (disconnection was defined either as death or liberation from mechanical ventilation without resumption of ventilatory support during index ICU admission)	0/193 (0%)
Vital status at time of disconnection from mechanical ventilation	0/193 (0%)
Vital status at time of discharge from hospital or day 90	2/193 (1%)[a]
Ventilator-free days at day 60	2/193 (1%)[a]
Duration of mechanical ventilation in ICU survivors	0/149 (0%)
Duration of ICU admission in ICU survivors	2/149 (1%)[b]
Duration of hospitalization in hospital survivors	2/126 (2%)[b]
Reintubation	0/193 (0%)
Tracheostomy	0/193 (0%)
Prolonged mechanical ventilation > 14 days	0/193 (0%)
Duration of weaning in patients in whom weaning was attempted	1/161 (1%)[c]

Reasons for missing data
[a]Two patients transferred out of study hospital after ICU discharge to another hospital prior to day 60 and unable to establish vital status in follow-up phone calls
[b]Two patients transferred from study ICU to the ICU of another hospital after liberation from mechanical ventilation; vital status at hospital discharge established in follow-up phone calls but unable to establish total duration of ICU admission or total duration of hospital admission
[c]Unable to locate date of first trial of spontaneous breathing

© 2020 Sklar MC et al. *JAMA Network Open.*

eTable 4
Patient characteristics according to baseline diaphragm thickness

Characteristics	Overall Study Population (n=193)	Baseline Diaphragm Thickness ≤2.3 mm (n=105)	>2.3 mm (n=88)	p-value
Age, year, mean (SD)	60 (15)	60 (16)	60 (13)	0.79
Female sex, n (%)	73 (38)	42 (40)	31 (35)	0.60
Height, cm	167 (162-176)	166 (160-177)	168 (163-175)	0.26
Weight, kg	76 (64-87)	72 (59-83)	77 (68-90)	0.01
Body mass index, kg/m^2	26.3 (22.4-30.4)	24.9 (22-29.6)	27.4 (23.4-30.7)	0.05
Immunocompromise*, n (%)	28 (15)	15 (15)	13 (15)	1.0
Location prior to index hospital admission				0.23
Home	154 (82%)	81 (80%)	73 (85%)	
Acute care facility	16 (9%)	12 (12%)	4 (5%)	
Chronic care facility	19 (10%)	10 (10%)	9 (11%)	
SAPS II	48 (35-58)	49 (35-58)	44 (34-57)	0.44
SOFA, mean over first 72h	10 (8-13)	10 (8-13)	10 (8-14)	0.82
Primary cause of acute respiratory failure, n (%)				0.64
Respiratory	59 (31)	36 (32)	27 (28)	
Cardiovascular	26 (14)	14 (12)	13 (13)	
Sepsis (non-pulmonary)	26 (14)	19 (17)	11 (11)	
Neurological	18 (9)	9 (8)	9 (9)	
Post-operative	20 (10)	10 (9)	13 (13)	
Transplantation	28 (15)	15 (13)	18 (19)	
Other	16 (8)	11 (10)	6 (6)	
Sepsis-3 criteria present in first 48h, n (%)	164 (85)	87 (83)	77 (88)	0.49
Baseline PaO_2/FiO_2 (mm Hg)	158 (105-233)	170 (109-233)	156 (104-236)	0.76
Positive end-expiratory pressure (cm H_2O)	8 (5-10)	8 (5-10)	8 (5-10)	0.28
Duration of hospitalization prior to intubation (days)	1 (0-3)	1 (0-3)	1 (0-3)	0.83
Time from intubation to baseline Tdi measurement (hours)	21 (14-26)	21 (14-26)	20 (14-27)	0.89
Charlson score, n	1 (1-4)	1 (1-4)	1 (0-3)	0.16
ILD, n (%)	22 (11)	13 (12)	9 (10)	0.79
COPD, n (%)	35 (18)	21 (20)	14 (16)	0.56
FEV1 % predicted (n=49)	54 (32-71)	51 (25-67.5)	54 (45.5-74.8)	0.22
FVC % predicted (n=49)	59 (42-82)	54 (34.5-80.5)	62.5 (47-82.8)	0.20
CHF, n (%)	19 (10)	12 (12)	7 (8)	0.56
Ejection fraction % (n=108)	60 (55-60)	60 (55-60)	60 (52-60)	0.64

Definition of abbreviations: CHF = congestive heart failure; COPD = chronic obstructive lung disease; FEV1 = forced expiratory lung volume in 1 second; FVC = forced vital capacity; ILD = interstitial lung disease; PaO_2/FiO_2 = ratio of arterial oxygen tension to fraction of inspired oxygen; PEEP = positive end-expiratory pressure; SAPS = Simplified Acute Physiology Score; SOFA = Sequential Organ Failure Assessment. All distributions are reported as median (interquartile range) unless otherwise noted.
*Defined as ongoing receipt of chemotherapy or radiation or daily use of steroids greater than or equal to the equivalent of prednisone 20 mg

© 2020 Sklar MC et al. *JAMA Network Open.*

eTable 5
Admission categories and diagnoses

Admission diagnosis category	Number admitted	Specific diagnoses (number)
**Cardiovascular**	26	Arrhythmia (1), Cardiac arrest (9), Cardiogenic shock (2), Heart failure (2), Hemorrhagic shock (2), Metabolic acidosis NYD (1), Myocardial infarction (1), Upper GI bleed (8)
**Neurological**	18	Coma NYD (1), Intracranial hemorrhage (non-SAH) (6), Status epilepticus (3), Stroke (4), Subarachnoid hemorrhage (3), Traumatic brain injury (1)
**Other**	16	Not available (1), Acute liver failure (2), Alcohol withdrawal (1), Aspirin overdose (1), Decompensated cirrhosis (4), Unknown drug overdose (1), Renal failure (5), Traumatic intra-abdominal injuries (1)
**Postoperative**	20	Post hepatectomy (2), Post nephrectomy (2), Post-operative respiratory failure (non-septic) (10), Post tracheal resection (1), Pulmonary endarterectomy (5)
**Respiratory**	59	ARDS (5), Aspiration (1), COPD exacerbation (6), Hypercapnia NYD (12), ILD exacerbation (1), Myasthenic crisis (1), Pneumonia (28), Pulmonary embolism (2), Pulmonary hemorrhage (1), Status asthmaticus (2)
**Sepsis**	26	Bowel obstruction (1), Cholangitis (1), Pancreatitis (1), Perforated viscous (1), Sepsis (non-pulmonary, undifferentiated) (20), Toxoplasmosis (1), Tuberculosis (1)
**Transplantation**	28	Post heart transplant (1), Post liver transplant (8), Post lung transplant (19)

© 2020 Sklar MC et al. *JAMA Network Open.*

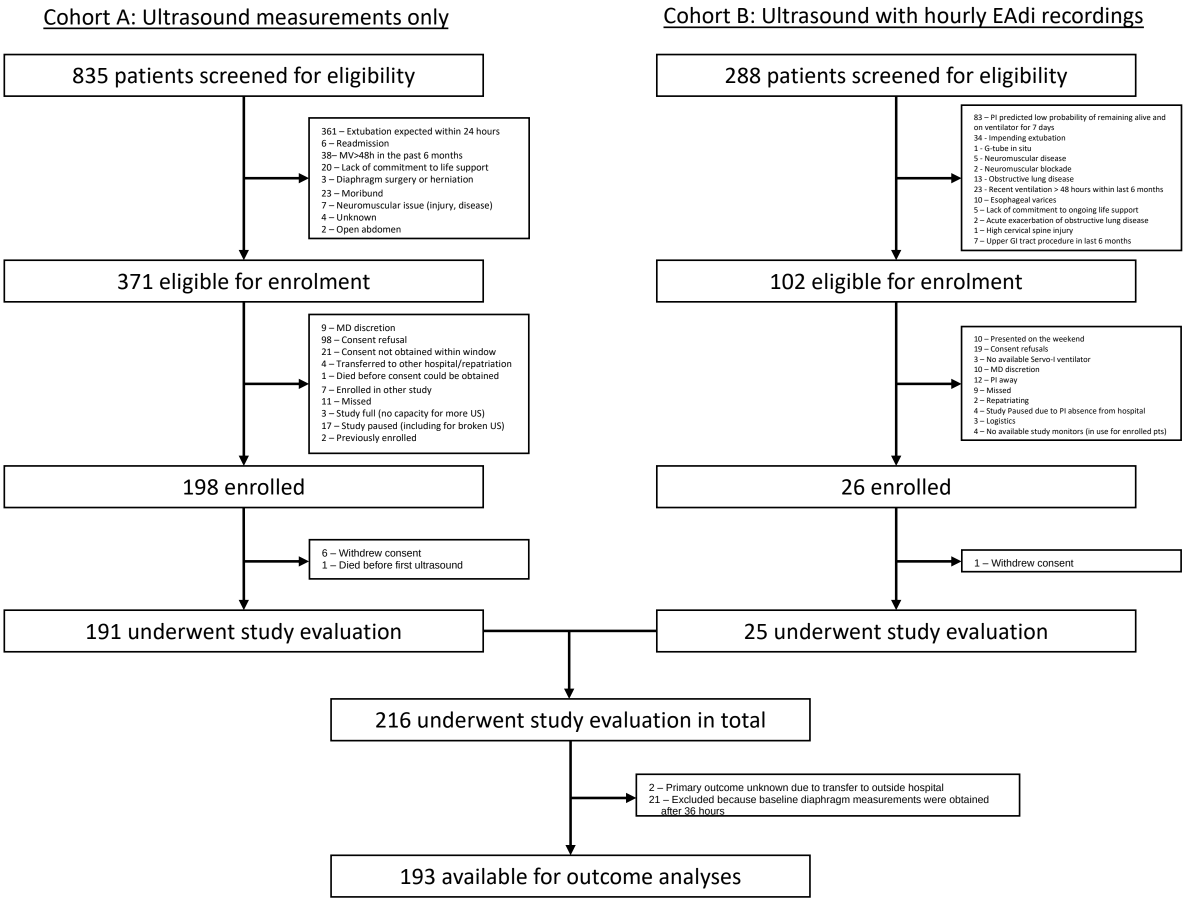

eFigure 1
CONSORT diagram and flow of study patients.

© 2020 Sklar MC et al. *JAMA Network Open.*

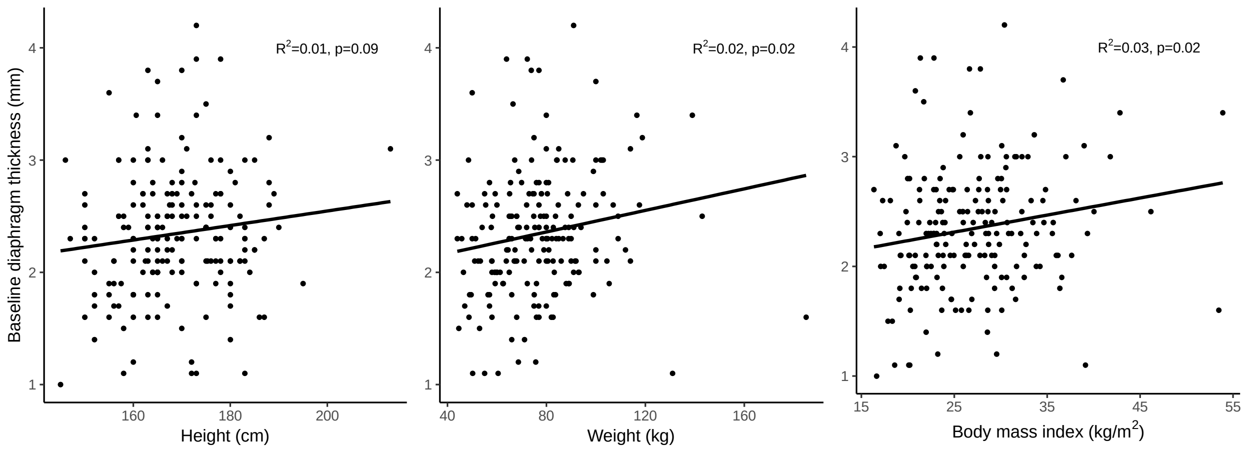

eFigure 2
Right hemidiaphragm thickness measured at the outset of mechanical ventilation was weakly correlated with patient height, weight, and body mass index.

© 2020 Sklar MC et al. *JAMA Network Open.*

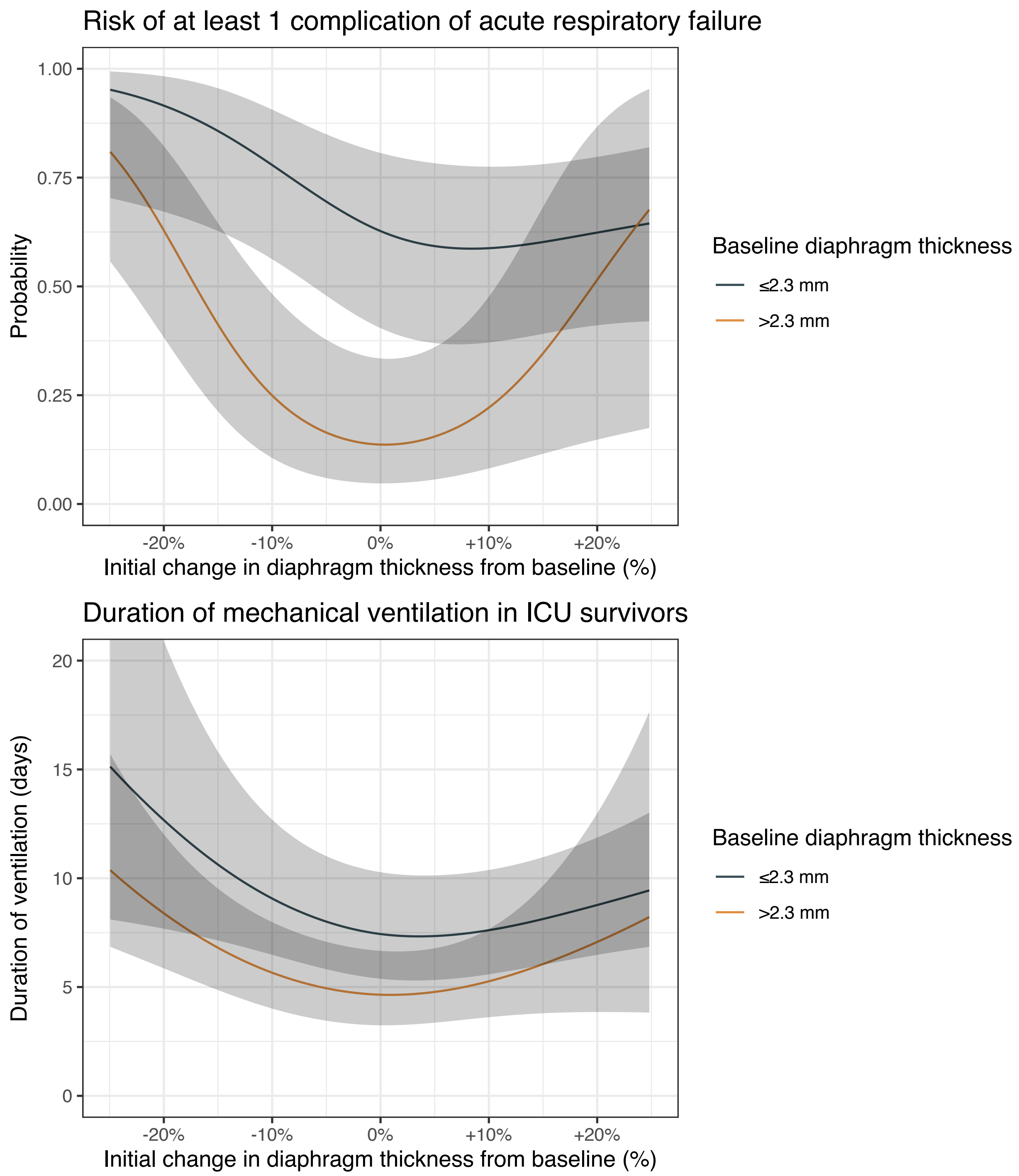

eFigure 3
The influence of baseline diaphragm thickness on the association between change in diaphragm thickness during mechanical ventilation and clinical outcomes (p=0.17 for interaction on risk of complications; p=0.75 for interaction on duration of ventilation).

© 2020 Sklar MC et al. *JAMA Network Open.*

eReferences

1. Riker RR, Picard JT, Fraser GL. Prospective evaluation of the Sedation-Agitation Scale for adult critically ill patients. *Crit Care Med*. 1999;27(7):1325-1329.

2. Yehya N, Harhay MO, Curley MAQ, Schoenfeld DA, Reeder RW. Reappraisal of Ventilator-Free Days in Critical Care Research. *Am J Respir Crit Care Med*. 2019;200(7):828-836. doi:10.1164/rccm.201810-2050CP

3. Unroe M, Kahn JM, Carson SS, et al. One-year trajectories of care and resource utilization for recipients of prolonged mechanical ventilation: a cohort study. *Ann Intern Med*. 2010;153(3):167-175. doi:10.7326/0003-4819-153-3-201008030-00007

4. Goligher EC, Dres M, Fan E, et al. Mechanical Ventilation–induced Diaphragm Atrophy Strongly Impacts Clinical Outcomes. *Am J Respir Crit Care Med*. 2017;197(2):204-213. doi:10.1164/rccm.201703-0536OC

© 2020 Sklar MC et al. *JAMA Network Open*.
